# Identifying Lung Cancer Cell Markers with Machine Learning Methods and Single-Cell RNA-Seq Data

**DOI:** 10.3390/life11090940

**Published:** 2021-09-09

**Authors:** Guo-Hua Huang, Yu-Hang Zhang, Lei Chen, You Li, Tao Huang, Yu-Dong Cai

**Affiliations:** 1 School of Life Sciences, Shanghai University, Shanghai 200444, China; 3280@hnsyu.edu.cn; 2 Department of Mechanical and Energy Engineering, Shaoyang University, Shaoyang 422000, China; 3468@hnsyu.edu.cn; 3 Channing Division of Network Medicine, Brigham and Women’s Hospital, Harvard Medical School, Boston, MA 02115, USA; reyhz@channing.harvard.edu; 4 Department of College of Information Engineering, Shanghai Maritime University, Shanghai 201306, China; lchen@shmtu.edu.cn; 5 CAS Key Laboratory of Tissue Microenvironment and Tumor, Shanghai Institute of Nutrition and Health, Chinese Academy of Sciences, Shanghai 200031, China

**Keywords:** lung cancer, random forest, decision tree, feature selection, cell biomarker, quantitative rules

## Abstract

Non-small cell lung cancer is a major lethal subtype of epithelial lung cancer, with high morbidity and mortality. The single-cell sequencing technique plays a key role in exploring the pathogenesis of non-small cell lung cancer. We proposed a computational method for distinguishing cell subtypes from the different pathological regions of non-small cell lung cancer on the basis of transcriptomic profiles, including a group of qualitative classification criteria (biomarkers) and various rules. The random forest classifier reached a Matthew’s correlation coefficient (MCC) of 0.922 by using 720 features, and the decision tree reached an MCC of 0.786 by using 1880 features. The obtained biomarkers and rules were analyzed in the end of this study.

## 1. Introduction

Non-small cell lung cancer is a major lethal subtype of epithelial lung cancer with high morbidity and mortality [1,2]. According to the epidemiological statistics from the American Society of Clinical Oncology [3], more than 84% of all clinical lung cancer cases can be attributed to non-small cell lung cancer. Annually, more than 116,300 men and 112,520 women in the United States alone are newly diagnosed with non-small cell lung cancer [4,5]. Owing to the development of pharmacological and clinical treatment techniques, the averaged death rate of non-small cell lung cancer has dropped by approximately 50%, especially in men [5]. However, the actual death rates vary with clinical background and disease stage. Therefore, exploring detailed pathological mechanisms and treatment strategies for this disease is necessary.

Previous studies on non-small cell lung cancer showed that genetic background and variations are major pathogenic factors for non-small cell lung cancer [6,7,8,9]. As driver genes, various pathogenic genes are specific and essential for the initiation and progression of the disease. For instance, the top identified gene associated with non-small cell lung cancer is EGFR. In 2004, the gene was identified as an effective clinical biomarker for the pathogenesis of non-small cell lung cancer [10]. Since then, various variation-based drugs (either targeted or chemotherapeutic drugs) have been developed, including necitumumab [11], cetuximab [12], and erlotinib [13]. Apart from EGFR, KRAS [14], EML4-ALK [15], ERBB2 [16], BRAF [17], and PIK3CA [18] participate in the pathogenesis of non-small cell lung cancer. Similar to EGFR, some of these typical genes have been used in developing novel drugs against non-small cell lung cancer, such as alpelisib targeting PIK3CCA, MK-2206 targeting AKT, and AZD6244 targeting MEK, confirming a complicated genetic background for the initiation and progression of non-small cell lung cancer.

Developments in sequencing techniques have stimulated research on non-small cell lung cancer at a single-cell level, and thus the internal heterogeneity of the disease has been revealed [19,20]. Single-cell RNA sequencing is one of the most widely applied and robust techniques for disease pathogenesis studies at the single-cell level [21]. By using single-cell RNA sequencing techniques, not only the transcriptomic heterogeneity of cancer cells but also different cell subgroups in microenvironments surrounding malignant tissues (cancer-adjacent tissues) can be further revealed [21], and thus research on non-small cell lung cancer has become extensive. In May 2020, researchers from Samsung Genome Institute applied systematic single-cell RNA sequencing to more than 0.2 million cells from 44 individuals to reveal specific and heterogeneous cell subgrouping patterns in pre-invasive and metastatic tumors and respective tumor microenvironments [22], providing novel systematic single-cell-level transcriptomic datasets of non-small cell lung cancers. The study mainly focused on showing differences among the transcriptomic profiles of different cell subtypes and among those of clinical pathological regions. However, key differentially expressed molecules (biomarkers) distinguishing different cells or cancers of different regions have not been fully revealed.

Here, to fulfill the research gap in identifying specific biomarkers for each cell subtype or pathological region, we divided transcriptomic data into 32 groups according to their tissue specificity, such as normal lung tissue (nLung), tumor lung tissue (tlung), brain metastatic tissue (mBrain), lymph node metastatic tissue (mLN), lymph node normal tissue (nLN), and pleural effusion (PE), and to cell subtypes, including B lymphocytes, endothelial cells, epithelial cells, fibroblasts, mast cells, myeloid cells, and T/NK cells. With advanced computational methods, we identified a group of qualitative classification criteria (biomarkers) distinguishing different cell subtypes from different pathological regions for the first time and established various rules for further quantitative distinction. Therefore, this study may not only confirm the applicability of computational methods on biomarker screening at the single-cell level but may also lay a solid foundation for further biomarker discovery on non-small cell lung cancer. 

## 2. Materials and Methods

We downloaded the processed single-cell RNA sequencing data of metastatic lung adenocarcinoma from Gene Expression Omnibus (GEO) under accession number GSE131907 [22]. This dataset included cells from mBrain, mLN, nLN, nLung, PE, and tLung. For each tissue, it included various cell types. We only considered the cell types with cell numbers greater than 100 for further analysis. In Table 1, we list the sample sizes of filtered cell types from six tissues. A total of 180,035 cells were obtained. In each cell, the expression levels of 29,634 genes were represented with normalized log2(TPM+1), as described by Kim et al. [22].

### 2.1. Boruta Feature Selection

Not all the adopted attributes were related to the target for most datasets. To remove or reduce redundancy between attributes, we employed the Boruta algorithm [23] for feature selection. The Boruta algorithm is a wrapper, using random forest [24] as a classifier. The basic idea behind such an algorithm is the removal of the most redundant feature each time. The Boruta algorithm is composed of the following steps: (1) shadow attributes are created by copying original attributes. (2) Each shadow attribute is shuffled for the removal of correlation with a target. (3) The shadow and the original attributes are used to train random forest and yield the importance of each attribute. (4) The maximum score is determined using the shadow attribute, and the original attribute whose score is more than the maximum score is marked as important. For each attribute with a score that is less than the maximum score, a two-sided test of equality is performed. (5) Unimportant attributes are removed. (6) The above steps are repeated until all the attributes are marked “important.” 

In this study, we used the Boruta program, available at https://github.com/scikit-learn-contrib/boruta_py (accessed on 5 March 2019). The single-cell RNA sequencing data of metastatic lung adenocarcinoma was fed into the program. Genes were termed as attributes and cell types were targets. Some genes marked as “important” were selected for further analysis.

### 2.2. Max-Relevance and Min-Redundancy

The Max-Relevance and Min-Redundancy (mRMR) proposed by Peng et al. [25] are filters for measuring correlations between attributes and targets on the basis of mutation information criteria. Mutation information between attribute *x* and target *y* is defined by:

(1) $$MI\left(x,y\right)=\overset{}{\underset{}{\iint }}p\left(x,y\right)log\frac{p\left(x,y\right)}{p\left(x\right)p\left(y\right)}dxdy,$$

where *p*(*x*, *y*) denotes the joint probabilistic density of *x* and *y*, and *p*(*x*) and *p*(*y*) denote the marginal probabilistic densities of *x* and *y*, respectively. The correlations of attribute *x* and target *y* increase with the value of *MI*(*x*, *y*). The mutation information between the set of attribute *S* and target is defined by:

(2) $$MI\left(S,y\right)=\frac{1}{\left|S\right|}\sum_{x\in S}^{}MI\left(x,y\right),$$

where the operator || denotes the number of elements in a set. The goal of the mRMR is to search a subset *S* from *n* attributes containing mutation information in which target *y* satisfies the maximum relevance and subset *S* satisfies the minimum redundancy. The max-relevance and min-redundancy is modeled by

(3) $$\underset{S}{\max}\left\{MI\left(S,y\right)-\frac{1}{{\left|S\right|}^{2}}\sum_{a,b∋S}^{}MI\left(a,b\right)\right\}$$

Equation (3) is an NP-hard question. The mRMR uses a greed strategy of searching; that is, it produces a list to sort attributes according to their importance.

This study adopted the mRMR program retrieved from http://penglab.janelia.org/proj/mRMR/ (accessed on 9 October 2017). Default parameters were used. The single-cell RNA sequencing data with genes selected by the Boruta method were fed into the mRMR program. Likewise, genes were termed as attributes and cell types were considered as targets. Accordingly, we obtained a gene list. Genes with high ranks were more important than those with low ranks.

### 2.3. Incremental Feature Selection

Incremental feature selection (IFS) [26] is a strategy for searching informative attributes. Owing to its simplicity and efficiency, IFS has been extensively used in feature selection. For the gene list obtained by the mRMR method, we added 10 genes each a time. That is, the decision tree and random forest first performed 10-fold cross-validation over the training set, in which cells were represented by the top 10 genes in the list, then those with top 20 genes, top 30 genes, and so on. When all genes were added, this procedure stopped. For each classification algorithm (decision tree or random forest), the gene set on which the classification algorithm provided the best performance was found. This set was called the optimum set and genes in this set were called optimum genes. 

### 2.4. Synthetic Minority Over-Sampling Technique

As listed in Table 1, the sizes of 32 cell types have great differences. The largest cell type contains much more cells than the smallest type. Thus, such a dataset is imbalanced. Classifiers built on such a dataset may be apt to the largest cell type. To solve such problem, we used the synthetic minority over-sampling technique (SMOTE) [27] to yield new samples for all cell types except the largest cell type. The SMOTE was described as follows. First, *k* nearest cells to one randomly selected cell of one minority cell type (cell type other than the largest cell type) were identified on the basis of Euclidean distance. Second, one nearest cell *b* was randomly selected from the above *k* nearest cells. The new cell *c* was computed by:

(4) c=a+rand(0,1)(b−a)

where rand(0,1) denotes a random number between 0 and 1. This newly produced cell was put into the minority cell type. After this procedure was performed several times, all cell types contained same number of cells. For convenience, the tool “SMOTE” in Weka (https://www.cs.waikato.ac.nz/ml/weka/ accessed on 15 December 2016) [28] was employed to tackle the imbalanced problem of single-cell RNA sequencing data.

### 2.5. Classification Algorithm

The IFS method needs one classification algorithm. This study selected decision tree [29] and random forest [24]. In a decision tree, each node represents a decision of one attribute. ID3, C5.0, and Classification and Regression Tree (CART) belong to the decision tree. CART with the Gini index was used in constructing a decision tree in this study. Random forest is an ensemble learning algorithm comprising many decision trees. Each decision tree is constructed by randomly selecting some attributes and some samples (with replacement). For a new sample, all the decision trees vote for a final decision.

In this study, we used the corresponding packages in Scikit-learn (https://scikit-learn.org/stable/ accessed on 26 August 2019) to implement the above decision tree and random forest. They were executed with their default parameters. The single-cell RNA sequencing data, where samples were represented by some genes, were fed into the program of decision tree or random forest to construct classifiers.

### 2.6. Cross-Validation and Metrics

We performed 10-fold cross-validation [30,31,32,33,34] to examine all classifiers in IFS. In 10-fold cross-validation, all cells were divided into 10 parts of equal or approximately equal size. Nine parts were used for training, and one part was used for testing. This process was repeated ten times. 

Matthew’s correlation coefficient (MCC) in multi-class [35] was mainly used in assessing performance, which was computed by:

(5) $$\mathrm{MCC}=\frac{cov\left(X,Y\right)}{\sqrt{cov\left(X,X\right)cov\left(Y,Y\right)}},$$

where *X* and *Y* are two matrices, representing the predicted and actual class of each sample. Similar to the original MCC for binary classification [36], such an MCC ranges from −1 to 1, and 1 means perfect prediction, 0 means random prediction, and −1 means completely opposite of prediction. 

In addition, we computed the accuracy of each cell type and overall accuracy for each classifier. These measurements were provided for reference. 

### 2.7. Functional Enrichment Analysis

With the IFS method, we can obtain optimum genes. To reveal their potential cell subtype specific biological functions, functional enrichment analysis was conducted. The results can be evidence for the further exploration of different contributions of different cell subtypes on lung tumorigenesis. In this study, we used R package topGO (v2.42.0) to perform gene ontology (GO) enrichment analyses. The *p*-value threshold for statistical significance was set to 0.001.

## 3. Results

**Figure 1:**
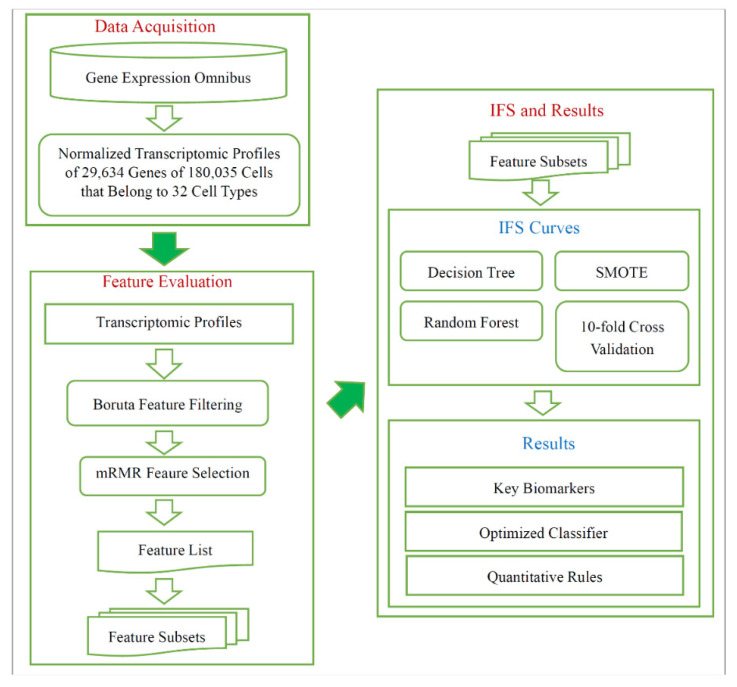
Flow chart illustrating the entire procedures. The single-cell RNA sequencing data are retrieved from Gene Expression Omnibus, which are analyzed by Boruta and mRMR methods. A feature list is generated, and it is fed into incremental feature selection (IFS), incorporating several methods (decision tree, random forest, SMOTE, 10-fold cross-validation), to extract key biomarkers, optimized classifier, and quantitative rules.

As shown in Figure 1, a computational procedure was used in investigating cell subtypes from the different pathological regions of non-small cell lung cancer. A total of 180,035 cells from 32 types were collected from GEO, and each cell had the expression levels of 29,634 genes. Then, Boruta feature selection and mRMR were performed successively. The 10-fold cross-validation was performed with the decision tree or random forest over the datasets generated by the SMOTE. We introduced results generated by all computational procedures.

### 3.1. Results of Boruta and mRMR Methods

We first applied the Boruta feature selection method to the single-cell RNA sequencing dataset. A total of 3880 genes were preserved, which are provided in Table S1. These genes were further analyzed by the mRMR method, yielding an ordered list of genes, which were ranked by importance (from highest to lowest). This list is also provided in Table S1. 

### 3.2. Results of IFS with Random Forest

**Figure 2:**
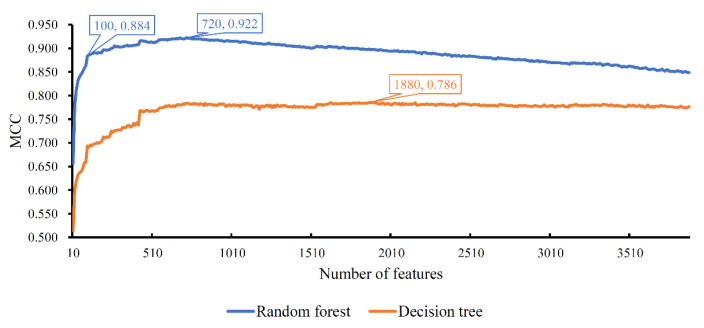
Curve of the incremental feature selection with different classification algorithms. Random forest yields the highest MCC (0.922) when top 720 features (genes) are used, whereas decision tree generates the highest MCC (0.786) when top 1880 features (genes) are adopted. Random forest with top 100 features (genes) also yields the high performance with MCC of 0.884, which can be a proper tool to identify cell types due to its higher efficiency compared with the random forest with top 720 features (genes).

**Figure 3:**
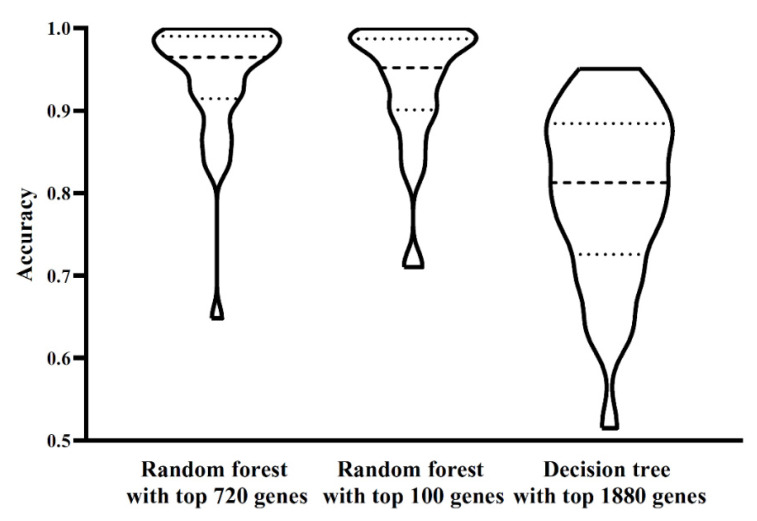
Performance of three classifiers on 32 cell types. The performance of two random forest classifiers is almost at the same level, whereas the decision tree classifier is inferior to two random forest classifiers.

The ordered list of genes was fed into the IFS method with random forest as the classification algorithm. We used step 10 to construct gene subsets and a random forest classifier was built on each gene subset. Each classifier was evaluated by 10-fold cross-validation. When evaluating the performance of classifiers, the SMOTE was used to balance samples from different cell types. The performance of each classifier, including accuracy for each cell type, overall accuracy and MCC, is available in Table S2. For an easy observation, an IFS curve was plotted, as shown in Figure 2, which set MCC as the *Y*-axis and the number of features (genes) as the *X*-axis. Evidently, random forest yielded the best MCC (0.922) with the top 720 features (genes). Accordingly, these top 720 genes constituted the optimum genes for random forest. The overall accuracy of such a classifier was 0.927 (see Table 2). The accuracy on each cell type was illustrated in a boxplot, as shown in Figure 3. These results indicated the good performance of this classifier. However, the efficiency of this classifier was not very high due to the large number of features (genes). By checking the curve in Figure 2 and MCCs in Table S2, random forest generates the MCC of 0.884 when the top 100 features (genes) were used. The overall accuracy was 0.891 (Table 2). They were a little lower than those of the best random forest classifier. As for the accuracy on each cell type, as shown in Figure 3, they were almost at the same level of those yielded by the best random forest classifier. Considering that much fewer features (genes) were used, this classifier was a more proper tool to identify cell types.

### 3.3. Results of IFS with Decision Tree

Although above random forest classifiers provided high performance, it is almost impossible to understand them and obtain new insights on the differences of cell types because they were absolute black-box classifiers. Thus, we further employed another classification algorithm, the decision tree, in the IFS method. This algorithm can learn a decision tree on a given dataset, from which several decision rules can be extracted. These rules can give a clearer picture on the differences of cell types. 

The IFS results on decision tree are provided in Table S2. Likewise, an IFS curve was also plotted, as illustrated in Figure 2. The best MCC was 0.786, which was obtained based on the top 1880 features (genes). The overall accuracy of this classifier was 0.799, listed in Table 2. Its performance on 32 cell types is shown in Figure 3. Evidently, the performance of such a classifier was much lower than the two above-mentioned random forest classifiers. However, it can give more insights. Accordingly, all cells, represented by the top 1880 features (genes), were learnt by the decision tree algorithm and a big tree was built. From such a tree, we extracted 19,032 decision rules, which are available in Table S3. Further analysis of these rules was helpful to uncover the differences of cell types.

Due to the huge number of rules, we tried to extract the most important information from these rules. For each cell type, the genes that only occurred in rules of such a cell type were extracted. We called them exclusive genes. Because these genes played critical roles for identifying a certain cell type, they can be latent biomarkers for some cell types. By counting the 19,032 rules, ten cell types received at least one exclusive gene, which are listed in Table 3. In Section 4.3, further analyses are conducted on them.

### 3.4. Enrichment Analysis on Essential Genes

**Figure 4:**
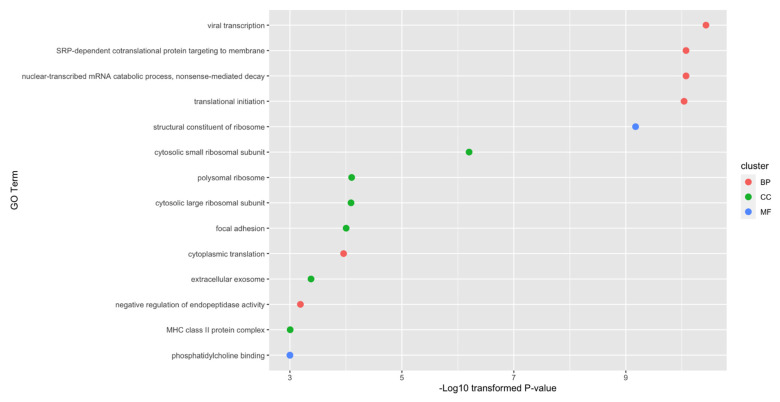
Gene ontology enrichment analysis on optimum genes. Fourteen gene ontology terms with *p*-value less than 0.001 are identified.

As mentioned in Section 3.2, the top 720 genes were the optimum genes for random forest. The enrichment analysis was conducted on them. As shown in Figure 4, 14 enriched GO terms with *p*-value threshold 0.001 were identified. Among these fourteen terms, there are six biological processes, six cellular components and two molecular functions. Among these enriched GO terms, specific functions such as viral transcription, focal adhesion and extracellular exosome have already been reported to be associated with specific cell types of lung cancer. The detailed discussion and interpretation of results can be seen in Section 4.4.

## 4. Discussion

We used our proposed computational methods to identify specific biomarkers or rules for distinguishing different cell subtypes from different tumor regions at the single-cell level in the primary and metastatic loci of non-small cell lung cancer. Recent publications have shown that several screened out qualitative biomarkers and quantitative rules contribute to the classification of 32 cell groups (six tissues and seven cell types) in the different stages and pathological regions of non-small cell lung cancer, implying the efficacy and accuracy of our proposed method. Detailed analyses on the correlations between non-small cell lung cancer and the top optimal biomarkers or rules are provided below.

### 4.1. Biomarkers for Tissue Specificity and Cell Subtype Classification

In our prediction list, we identified multiple biomarkers that contribute to the distinction of different cell types from the different regions of non-small cell lung cancers. Genes in such a list have been confirmed to distinguish at least two groups of cells according to recent publications. Here, we chose the top genes for detailed analyses as outlined below.

The first gene in our prediction list turned out to be *TYROBP*, which encodes an effective immune signaling adapter. According to previous studies, this gene has different expression patterns in different non-small cell lung cancer tissues. In early 2004, *TYROBP* was shown to be differentially expressed in tumor tissues in contrast in normal tissues [37], indicating that the gene may help distinguish tumor cells from normal cells. In May 2020, another study revealed that *TYROBP* was found to be one of the most significant biomarkers reflecting the immune status of tumor microenvironments and contributing to the distinction of functional and dysfunctional immune cells [38]. Therefore, the gene may play a role in distinguishing between normal and tumorous lymph node tissues because of differences in immune characteristics under physical or pathological (tumorigenic) conditions. As for the applications, such a gene can identify lung cancer using the biopsy sample directly from lung tissue, acting as additional lung tissue tumorigenesis monitoring biomarkers.

Apart from *TYROBP*, several candidate biomarkers encode functional Cluster of Differentiation (CD) molecules and contribute to the classification of different immune cells during the initiation and progression of non-small cell lung cancer. Hence, they are key immune biomarkers distinguishing immune cells under different conditions. *CD52*, as the top optimal CD molecule, is differentially expressed in the activated T cells compared with that in T cells that are not activated (in normal tissues) or inhibited. Therefore, the expression level of the *CD52* gene may be used in distinguishing T cells from different environments, validating the efficacy and accuracy of our prediction [39]. Further studies on immune cells in multiple non-small cell lung cancer confirmed that the gene is differentially expressed in tumor tissues unlike in normal tissues [40], consistent with our speculation. Apart from *CD52*, another gene in our prediction list from the CD family was *CD3D*, which encodes the delta of the CD3 complex of the T-cell receptor and is involved in T cell development [41] and T cell-mediated immune response [42,43]. Similar to *CD52*, *CD3D* is differentially expressed in the normal and malignant lung tissues of non-small cell lung cancer [44], consistent with our prediction. A systematic study [20] published in 2018 investigated T cell expression pattern in non-small cell lung cancer at the single-cell level, which confirmed that *CD3D* has differential expression levels not only in normal tissues and malignant tissues but also in the lung tissues and lymph nodes, implying that the gene is a potential biomarker distinguishing T cells from different tissues during the initiation and progression of non-small cell lung cancers. Such a cluster of differentiation proteins can monitor the immune status of the tumor microenvironment at the single-cell level. Theses biomarkers can provide valuable information for the comprehensive immune evaluation of tumor patients in clinic.

Apart from the two clusters of differentiation biomarkers, *CD79A* as a key biomarker for B cells [45] and *CD37* as a significant regulator for T–B interactions [46] are effective biomarkers identified by our proposed computational methods. According to recent publications, *CD79A* has a specific expression level in carcinoma-associated fibroblasts in non-small cell lung cancers [47]. Therefore, considering the gene a potential biomarker for subgrouping non-small cell lung cancer-associated cells is reasonable, especially tumor-associated fibroblasts. As for *CD37*, an independent study, published in 2019, on the brain metastasis of non-small cell lung cancer identified *CD37* as an effective biomarker for lung cancer brain metastasis [48], consistent with our prediction of *CD37* as a potential biomarker for non-small cell lung cancer-associated cell subgrouping. Therefore, our predicted genes, such as *CD79A* and *CD37*, definitely contributed to the subgrouping of the 32 candidate groups of cells with different cell types and tissue specificity and proved at least useful in identifying specific cell subtypes, such as fibroblasts, in tumor tissues and brain metastatic tumor tissues. Similar with *CD3D* and *CD52*, *CD79A* and *CD37* can not only help us monitor specific T–B interactions, which is one of the major parts of the anti-cancer immune response, but can also provide us a new biomarker to evaluate the alteration of cancer microenvironment during tumorigenesis.

The next predicted gene in our prediction list, *HLA-DRA*, is also associated with the immune system. As one of the major genes involved in immune recognition processes, it is mainly expressed in antigen-presenting cells, including B cells [49], mast cells [50], and fibroblasts [51]. Therefore, its expression level can be used in distinguishing the three groups of cells from other cells regardless of tissue specificity. As for the distinctive potential of the gene in non-small cell lung cancer, researchers from MD Anderson Cancer reported in 2018 that *HLA-DRA* has different expression levels in the normal and tumor tissue sections in clinical FFPE samples [52]. Therefore, our predicted gene *HLA-DRA* can also be an effective biomarker distinguishing different cell subtypes from different tissues in non-small cell lung cancers. *HLA-DRA* is a potential biomarker that can be used to evaluate the antigen-presenting efficacy for cancer monitoring or immune therapy effectiveness evaluation.

Apart from the genes described above, other genes, such as *SRGN*, which mediates specific mast cell-related granules secretion [53], *S100A11*, which mediates leukocyte differentiation [54], *GPX1*, which protects hemoglobin in circulating systems [55], and *FCER1G*, which regulates IgE-mediated immune response [56] in mast cells, contribute to the identification of specific subtypes of cells in certain tissues in non-small cell lung cancers. Moreover, *SRGN* [57], *S100A11* [58], and *FCER1G* [59] contribute to the identification of mast cells in tumor microenvironments. This finding is consistent with our prediction on their specific capacities on cell subgrouping. As for *GPX1*, it has a specific expression level in the brain [55] and may thus be useful in distinguishing the cells of brain metastasis from other cell subtypes. These features validated our prediction. Such genes can also be used as potential biomarkers to monitor abnormal alteration during the initiation, progression and metastasis of lung cancer.

In summary, the optimal features or genes in our prediction list are definitely useful in qualitatively subgrouping the non-small cell lung cancer-associated cells of different cell subtypes and tissue specificity. They represent the expression profiling characteristics of their respective cell types from different tissues. As for their application, they can be used as potential biomarkers to evaluate and monitor the abnormal proliferation, metastasis or cell type transformation for each cell subtype, which may be quite important and valuable for the clinical diagnosis and therapy of lung cancers and their progression. Thus, they can be candidate lung cancer biomarkers marking different stages, regions and cell groups of malignant lung cancers. This finding also indicated the efficacy of our proposed computational methods.

### 4.2. Rules for Quantitative Tissue Specificity and Cell Subtype Classification

Apart from qualitative genes, we set up a group of quantitative rules for the accurate classification of different cell subtypes and tumorigenic regions of non-small lung cancers. All the parameters from the rules were correlated with single-cell level classification and showed corresponding expression tendency with the predicted rules, validating the efficacy and accuracy of our prediction. Here, we selected the most typical rule for each class of cells for further discussion. All the rules are listed in Table S3. Detailed analyses are shown below.

The first seven classes of cells were derived from the brain metastasis of non-small cell lung cancer. According to our predicted rules for Class 1–7, the seven groups of rules shared a specific expression pattern, that is, the low expression level of *TPSB2*, except rule 4, which described the fibroblasts of the brain metastasis. As for the other six cell subtypes, previous studies showed that a low *TPSB2* expression level is one of the typical expression patterns in cells from the brain metastasis of non-small cell lung cancer [60], implying the accuracy and efficacy of our predicted rules. As for the fibroblasts in brain metastasis, *TPSB2* expression is upregulated in fibroblasts from inflammatory environments [61], corresponding to the microenvironment of metastases. As for cell type specificity distinction, *B2M* expression is upregulated in B cell subgroups, which can help identify cells from class 1 (rule 687, mBrain_B lymphocytes) [62]. *PLPP2* expression is downregulated in the endothelial cells of the brain [63], contributing to the identification of cells from class 2 (rule 6292, mBrain_Endothelial cells). As for class 3 (rule 0, mBrain_Epithelial cells), another parameter named *DCN* is downregulated in epithelial cells. Given that *DCN* [64] is downregulated in the epithelial cells of brain tissues, considering it a quantitative parameter is reasonable. Class 4 (rule 72) described the fibroblasts with a specific expression pattern (low expression level of *SFTPC*). The low expression level and extremely high level of the gene are correlated with the brain metastases of malignant lung diseases, including non-small cell lung cancer [65], validating the efficacy and accuracy of our prediction. As for class 5–7, contributing to the identification of specific cell types mast cells, myeloid cells and T/NK cells, specific quantitative biomarkers with specific expression tendencies, such as a high *SPP1* expression level (rule 678) for mast cells [66], a low *TRAC* expression level (rule 700) for myeloid cells [67], and a high CD79A expression level (rule 883) for T/NK cells [47,68] contribute to the establishment of immune microenvironments under certain pathological conditions, including the pathogenesis of non-small cell lung cancers, validating the efficacy and accuracy of our prediction.

As for the next seven classes of cell subtypes contributing to the identification of normal or metastatic lymph node-associated cells, the rules of such a group all contained a specific biomarker with *IFI44L*, which has a specific high expression level in metastatic lymph nodes and a relatively low expression level in normal lymph nodes (rule 16 and 53), corresponding with recent publications on the potential clinical predictive capacities of the gene [69]. As for the detailed cell subtypes, similar with cells in brain metastasis, metastatic B cells, epithelial cells, myeloid cells and T/NK cells also have specific biomarkers with similar expression tendencies to *TGM2* (rule 35) [70,71], *TYROBP* (rule 82) [72], *TRAC* (rule 94) [73], and *CD79A* (rule 156) [74] confirmed in the lymph node under pathological/malignant conditions with corresponding variation trends. As for the normal controls, we found a group of specific biomarkers with expression tendencies confirmed by recent publications. Class 10 normal lymph nodes have a specific expression level of *CD79A*, marking different groups of B cells [46]. In rule 8, the high level of the gene indicated the target cell as B cells from lymph nodes, validating the efficacy and accuracy of our prediction. In normal lymph nodes, the specific expression level of *RPS27* (upregulated, rule 53) [75] and *TRAC* (rule 75) [76] shared similar expression levels with tumor comparison [77], and *SFTPC* (rule 174) [76] was involved in the identification of specific cell groups: B lymphocytes, myeloid cells, and T/NK cells, validating the efficacy and accuracy of our prediction.

For the next 14 classes: class 15-21, class26-32, various cell subgroups from lung tumor tissues, and normal tissues were gradually analyzed, and specific quantitative biomarkers were identified for each group. For each cell subgroup with specific tissue specificity, we identified some optimal biomarkers for accurate subgrouping. In our prediction rules for normal lung tissues, *ITGA5* (rule 167), *MT-ND2* (rule 45), *SFTPC* (rule 114), *ADH1B* (rule 21), *MMP7* (rule 60), *TYROBP* (rule 92), and *SYK* (rule 71) are typical biomarkers for seven clusters of lung normal cells (B lymphocytes [78], endothelial cells [79], epithelial cells [80], fibroblasts [81], mast cells [82], myeloid cells, and T/NK cells [83]), which had specific expression tendencies and had been validated by recent publications. As for the malignant tissues, for each of the seven cell subgroups of lung malignant tissues in non-small cell lung cancer, *IGLC2* (rule 17), *AXL* (rule 189), *SERPINA1* (rule 190), *CALD1* (rule 265), *APOE* (rule 383), *S100A8* (rule 393), and *GSN* (rule 405) are useful in distinguishing the following cell subgroups: B lymphocytes [84], endothelial cells [85], epithelial cells [86], fibroblasts [87], mast cells [88], myeloid cells [89], and T/NK cells [90]. The correlations between gene expression levels and cell subgrouping were confirmed by recent publications, validating the prediction efficacy of our newly presented computational method.

As for the remaining four classes describing pleural effusion-associated cells, rule-predicted cells from the four classes were validated by recent publications. For the first subgroup of cells from pleural effusion, the B lymphocytes, recent publications confirmed that one of our predicted upregulated parameters, *TPT1* (rule 47), can definitely contribute to the identification of B lymphocytes in samples obtained from pleural effusion [91,92], validating the efficacy and accuracy of our prediction. Similarly, the upregulated genes, *APRT* in rule 138, *S100A8* in rule 14, and *GIMAP7* in rule 90, are all typical biomarkers for the identification of specific cell subtypes: epithelial cells, myeloid cells, and T/NK cells, which are further supported by recent publications [93,94,95,96].

### 4.3. Analysis of Exclusive Genes

As one of the main results of this study, several quantitative rules to distinguish cell types were established. We extracted exclusive genes for each cell type as described in Section 3.3. Ten cell types had their own exclusive genes, which are listed in Table 3. They may provide their specific contributions on their respective cell types. To reveal their linkage to the corresponding cell types, we conducted an extensive discussion on them.

Two genes, *C5orf38* and *MLLT4*, have been shown to be specific biomarkers for lung cancer epithelial cells in situ. *C5orf38* has been shown to be associated with multiple epithelial tumorigenesis [97,98], implying its specific expression pattern in lung cancer epithelial cells compared to other cell types. As for *MLLT4*, this gene has also been reported as a risk factor for lung epithelial tumorigenesis [99], validating such a result. Using such two biomarkers, it is easy to identify whether lung epithelial cells have been malignantly transformed or not.

Three genes, *P4HA2*, *TJP3*, and *BAIAP2L1*, have been shown to be associated with lung cancer B lymphocytes in situ. *P4HA2* has been reported to be an extracellular matrix signaling-associated gene and was shown to participate in B-cell-mediated immune responses [100]. As for *TJP3*, although no direct evidence has connected such a gene with lung cancer B cells, it has been reported to participate in the regulation of antibody-mediated immune responses during lung tumorigenesis [101], validating such a result. Another gene, *BAIAP2L1*, is a general regulator associated with the tumor microenvironment [102], which may identify lung cancer B lymphocytes.

Three genes, *TFAP2A*, *TJP1* and *TMEM63B*, have been shown to be associated with the B lymphocytes in lymph node metastasis of lung cancer. *TFAP2A* [103] and *TJP1* [104] have both been identified in the lymph node of lung adenocarcinoma as potential biomarkers. As for *TMEM63B*, it has also been shown to be associated with immune responses during the metastasis of lung cancer [105], associated with the summarized cell subtype.

As for other clusters, *RAB40B* and *SLC9A3R2* have been shown to be associated with T/NK cells in the lymph node metastasis of lung cancer [106]. *TMEM45B* has been shown to be associated with myeloid cells in lymph node metastasis of lung cancer [107]. *TM7SF2* has been shown to be associated with T/NK cells in normal lymph nodes [103]. *ZDHHC9* has been shown to be associated with myeloid cells in pleural effusion [108]. *GNG12* and *ITGA2* have been reported to relate to T/NK cells in pleural effusion [109]. *FNBP1L* and *PTPN13* have been shown to be related to endothelial cells of lung tumors [110]. *FAM83H* has been shown to be associated with the T/NK cells in brain metastasis of lung cancer [109]. All these findings validated our results.

### 4.4. Functional Enrichment Results on Optimum Genes

As mentioned in Section 3.4, 14 enriched GO terms with *p*-values less than 0.001 were identified. All such GO terms were shown to be associated with cell-subgroup-specific contribution to lung tumorigenesis, validating the idea that the optimum genes can distinguish different cell subtypes and reveal their potential biological mechanisms. The detailed discussion on significant GO terms can be seen below.

Due to the limitation of the length of the manuscript, we selected three typical GO terms with the lowest *p*-value from each GO cluster. They were viral transcription (GO:0019083) for biological processes, the cytosolic small ribosomal subunit (GO:0022627) for cellular components and the structural constituent of ribosome (GO:0003735) for molecular functions.

According to recent publications, viral infections and transcriptions have been shown to be associated with the metastasis of lung cancer [111]. Thus, it is reasonable for the optimum genes to be enriched in the viral-transcription-associated biological processes. Specifically, viral infections have already been confirmed in multiple cancer subtypes to be associated with different kinds of malignant metastasis [112,113]. Other biological process terms may also be linked to the tumorigenesis of lung cancer in their respective ways. For the cellular component cytosolic small ribosomal subunit, it has been shown to be abnormally regulated during the initiation and metastasis of lung cancer [114], corresponding with our results. As for the structural constituent of ribosome, in the same paper [114], authors also validated the specific role of ribosome during lung cancer tumorigenesis.

## 5. Conclusions

We presented a random forest- and decision tree-based method for classifying cell subtypes from different pathological regions of non-small cell lung cancer. The selected the qualitative features (genes) and quantitative rules were confirmed to be correlated with non-small cell lung cancer and involved in the complicated classification of different cell subtypes. Therefore, the identified genes and rules can be potential biomarkers for the clinical diagnosis and monitoring of non-small cell lung cancer targeting different cell subtypes and tumorigenic focus, enriching techniques for the clinical treatment of non-small cell lung cancer. The key codes are available in Code S1.

## Figures and Tables

**Table 1:** The sample sizes of different cell types.

Tag	Cell Types	Sample Size
Class 1	mBrain B lymphocytes	1311
Class 2	mBrain Endothelial cells	159
Class 3	mBrain Epithelial cells	15,463
Class 4	mBrain Fibroblasts	444
Class 5	mBrain MAST cells	508
Class 6	mBrain Myeloid cells	5657
Class 7	mBrain T/NK cells	2683
Class 8	mLN B lymphocytes	6062
Class 9	mLN Epithelial cells	3053
Class 10	mLN Myeloid cells	5442
Class 11	mLN T/NK cells	5069
Class 12	nLN B lymphocytes	10,584
Class 13	nLN Myeloid cells	1288
Class 14	nLN T/NK cells	19,478
Class 15	nLung B lymphocytes	634
Class 16	nLung Endothelial cells	1295
Class 17	nLung Epithelial cells	3703
Class 18	nLung Fibroblasts	1585
Class 19	nLung MAST cells	1079
Class 20	nLung Myeloid cells	16,672
Class 21	nLung T/NK cells	11,413
Class 22	PE B lymphocytes	3285
Class 23	PE Epithelial cells	396
Class 24	PE Myeloid cells	3063
Class 25	PE T/NK cells	9192
Class 26	tLung B lymphocytes	5781
Class 27	tLung Endothelial cells	645
Class 28	tLung Epithelial cells	13,852
Class 29	tLung Fibroblasts	1739
Class 30	tLung MAST cells	1809
Class 31	tLung Myeloid cells	10,123
Class 32	tLung T/NK cells	16,568

**Table 2:** Performance of random forest and decision tree with some top features.

Classification Algorithm	Number of Features	Overall Accuracy	MCC
Random forest	720	0.927	0.922
Random forest	100	0.891	0.884
Decision tree	1880	0.799	0.786

**Table 3:** Exclusive genes for some cell types.

Tag	Cell Type	Exclusive Gene ^a^
Class 7	mBrain T/NK cells	FAM83H
Class 8	mLN B lymphocytes	TFAP2A, TJP1, TMEM63B
Class 10	mLN Myeloid cells	TMEM45B
Class 11	mLN T/NK cells	RAB40B, SLC9A3R2
Class 14	nLN T/NK cells	TM7SF2
Class 24	PE Myeloid cells	ZDHHC9
Class 25	PE T/NK cells	GNG12, ITGA2
Class 26	tLung B lymphocytes	P4HA2, TJP3, BAIAP2L1
Class 27	tLung Endothelial cells	FNBP1L, PTPN13,
Class 28	tLung Epithelial cells	C5orf38, MLLT4

^a^: Exclusive gene for one cell type is defined as the genes that only occur in rules for such a cell type.

## Data Availability

The data presented in this study are openly available in Gene Expression Omnibus at https://www.ncbi.nlm.nih.gov/geo/query/acc.cgi?acc=GSE131907, reference number [22].

## Supplementary Materials

The following are available online at https://www.mdpi.com/article/10.3390/life11090940/s1, Table S1: feature list yielded by the mRMR method, Table S2: performance of two classification algorithms on different feature subsets, Table S3: rules extracted from decision tree. Code S1: key codes used in this study.
