# Supplementary material for: Identifying Lung Cancer Cell Markers with Machine Learning Methods and Single-Cell RNA-Seq Data
Source: Life (Basel). 2021 Sep 9;11(9):940. doi: 10.3390/life11090940 (PMC8467493; doi:10.3390/life11090940)
Supplement: Supplementary file 1 [file life-11-00940-s001.zip › life-1315588-supplementary/life-1315588-sup/Code S1.zip!/Code S1/Read me.pdf]

Guide for codes

1. Package dependencies

- scikit-learn 0.23 and later
- imbalanced-learn v0.6.2
- pandas
- Python 3.6 or newer

2. OS requirements

This package is supported for Linux/macOS/Windows 10. The package has been tested on the following systems:

- Linux: Ubuntu 20.04 LTS/CentOS 7.9
- Windows: Windows 10

3. Train a random forest with SMOTE based on scikit-learn

python sk-rf-kfold-smote-run.py --datapath inputfile -t 100 -d 1000 -e 100 -r 88 -k 10 -n 2

- -t: Number of trees in the forest.
- -d: The maximum depth of the tree. If none, nodes are expanded until all leaves are pure or until all leaves contain less than min_samples_split samples.
- -e: Number of training epochs.
- -k: Number of folds. Must be at least 2.
- -r: Pseudo-random number generator state used for shuffling.
- -n: Number of nearest neighbors to construct synthetic samples.
- --datapath: The path of dataset.

4. Train a decision tree with SMOTE based on scikit-learn

python sk-dt-smote-kfold-run.py -k 10 --datapath inputfile

- -k: Number of folds. Must be at least 2.
- --datapath: The path of dataset.

5. Note for utils.py

This package is for counting some measurements and called in sk-rf-kfold-smote-run.py and sk-dt-smote-kfold-run.py.
